# Formation of singlet oxygen by decomposition of protein hydroperoxide in photosystem II

**DOI:** 10.1371/journal.pone.0181732

**Published:** 2017-07-21

**Authors:** Vinay Pathak, Ankush Prasad, Pavel Pospíšil

**Affiliations:** Department of Biophysics, Centre of the Region Haná for Biotechnological and Agricultural Research, Faculty of Science, Palacký University, Olomouc, Czech Republic; University of Hyderabad School of Life Sciences, INDIA

## Abstract

Singlet oxygen (^1^O_2_) is formed by triplet-triplet energy transfer from triplet chlorophyll to O_2_ via Type II photosensitization reaction in photosystem II (PSII). Formation of triplet chlorophyll is associated with the change in spin state of the excited electron and recombination of triplet radical pair in the PSII antenna complex and reaction center, respectively. Here, we have provided evidence for the formation of ^1^O_2_ by decomposition of protein hydroperoxide in PSII membranes deprived of Mn_4_O_5_Ca complex. Protein hydroperoxide is formed by protein oxidation initiated by highly oxidizing chlorophyll cation radical and hydroxyl radical formed by Type I photosensitization reaction. Under highly oxidizing conditions, protein hydroperoxide is oxidized to protein peroxyl radical which either cyclizes to dioxetane or recombines with another protein peroxyl radical to tetroxide. These highly unstable intermediates decompose to triplet carbonyls which transfer energy to O_2_ forming ^1^O_2_. Data presented in this study show for the first time that ^1^O_2_ is formed by decomposition of protein hydroperoxide in PSII membranes deprived of Mn_4_O_5_Ca complex.

## Introduction

Photosystem II (PSII), a pigment-protein complex present in the thylakoid membrane, catalyzes the reduction of plastoquinol and oxidation of water [1–3]. The redox process is driven by light energy absorbed by chlorophyll in the PSII antenna complexes which is transferred from trimeric light-harvesting complex II (LHCII) to the inner chlorophyll antenna (CP43 and CP47) and to the PSII reaction center (D1/D2/cytochrome *b*_559_) [4, 5]. Charge separation between the chlorophyll monomer (Chl_D1_) and pheophytin (Pheo_D1_) of D1 protein forms ^1^[Chl_D1_^•+^Pheo_D1_^•−^] radical pair which is fast stabilized by the oxidation of the weakly-coupled chlorophyll dimer P_D1_ and P_D1_ (P680) forming ^1^[P680^•+^Pheo_D1_^•−^] radical pair [6–8]. Reduction of P680^•+^ by a redox-active tyrosine residue (TyrZ) forms tyrosyl radical (TyrZ^•^). Oxidizing TyrZ^•^ and reducing Pheo^•−^ equivalents are used for the oxidation of water and the reduction of plastoquinone on the lumenal and stromal side of the thylakoid membrane, respectively.

Water oxidation is catalyzed by Mn_4_O_5_Ca complex which consists of four oxo-bridged Mn atoms and one Ca atom [9–12]. When Mn_4_O_5_Ca complex is absent (photoactivation, photoinactivation), TyrZ^•^ and P680^•+^ are not reduced by an electron from Mn_4_O_5_Ca complex [13, 14]. Under these circumstances, the lipids and proteins located nearby highly oxidizing TyrZ^•^ and P680^•+^ are oxidized forming lipid (L^•^) and protein (P^•^) radicals. Using EPR spin trapping spectroscopy, it was demonstrated that exposure of PSII membranes deprived of Mn_4_O_5_Ca complex forms L^•^ [15]. Reaction of L^•^ and P^•^ with O_2_ forms lipid (LOO^•^) and protein (POO^•^) peroxyl radicals. It was demonstrated that the reaction of L^•^ with O_2_ as monitored by O_2_ photoconsumption was suppressed by exogenous electron donor confirming the involvement of TyrZ^•^ in this process [16]. Additional hydrogen abstraction from lipids and proteins by LOO^•^ and POO^•^ forms lipid (LOOH) and protein (POOH) hydroperoxides, respectively [17, 18]. Using fluorescence spectroscopy, it was demonstrated that exposure of PSII membranes deprived of Mn_4_O_5_Ca complex to high light forms LOOH and POOH [19]. It was evidenced that POOH is less abundant (4 molecules per PSII reaction center), while LOOH is more abundant (200 molecules per PSII reaction center). More recently, it has been evidenced that LOOH and POOH might play a crucial role in PSII damage on the PSII electron donor side [20].

Singlet oxygen is formed by the triplet-triplet energy transfer from triplet chlorophyll (^3^Chl*) to O_2_ by Type II photosensitization reaction [21–24]. In the PSII antenna complex, ^3^Chl* is formed by a change in spin state of the excited electron via intersystem crossing. The probability of the intersystem crossing is increased, when excess excitation energy is not effectively utilized for photochemistry or deactivated by carotenoid quenching. In the PSII reaction center, ^3^P680* is generated by the charge recombination of triplet radical pair ^3^[P680^•+^Pheo^•−^] formed by the change in spin orientation of the primary radical pair ^1^[P680^•+^Pheo^•−^]. Apart from ^3^Chl*, ^1^O_2_ is formed by triplet-triplet energy transfer from lipid triplet carbonyl (^3^L = O*) to O_2_ [25–27]. Lipid triplet carbonyls are formed by the decomposition of high energy intermediates lipid dioxetane (LOOL) and lipid tetroxide (LOOOOL) formed by the cyclization of LOO^•^ and by the recombination of LOO^•^, respectively. Alternatively, ^1^O_2_ is formed directly by decomposition of tetroxide via Russell mechanism [28]. It was demonstrated that ^1^O_2_ is formed by decomposition of high energy intermediates under various types of environmental stresses (high light, heat, heavy metals, wounding) [25, 26, 29]. Using EPR spin trapping spectroscopy, it was demonstrated that exposure of PSII membranes deprived of Mn_4_O_5_Ca complex to high light results in ^1^O_2_ formation [15]. Based on the observation that ^1^O_2_ formation correlates with L^•^ formation, the authors proposed that recombination of LOO^•^ via Russell mechanism might be responsible for ^1^O_2_ formation. Although ^1^O_2_ formation by lipid peroxidation has been described to some extent, there is no evidence provided on the ^1^O_2_ formation by protein oxidation.

In this study, evidence has been provided for the first time to show that ^1^O_2_ is formed by decomposition of POOH formed by protein oxidation in PSII membranes deprived of Mn_4_O_5_Ca complex. The protein oxidation is initiated by highly oxidizing species formed by charge separation of excited chlorophylls (by Type I photosensitization reaction) both in the PSII antenna complex and the PSII reaction center. It is proposed that POOH decomposes to protein dioxetane (POOP) and protein tetroxide (POOOOP). These high-energy intermediates decompose to ^3^P = O* which transfers excitation energy to O_2_ forming ^1^O_2_.

## Materials and methods

### PSII membranes

PSII membranes were isolated from fresh spinach (*Spinacia oleracea*) leaves using a procedure described by Berthold and co-workers [30]. For removal of Mn_4_O_5_Ca complex, PSII membranes (1 mg ml^-1^) were treated with 0.8 M Tris-HCl (pH 8) for 30 min in the dark. Tris-treated PSII membranes were stored at −80°C in the dark for further use. LHCII were separated by ultracentrifugation on a sucrose gradientusing a protocol from Caffarri and co-workers [31] with some modifications. Briefly, PSII membranes (200 μg Chl) were washed once with 10 mM HEPES pH 7.5 and solubilized by adding an equal volume of 0.6% α-dodecylmaltoside (α-DM) in 10 mM HEPES pH 7.5 and vortexing for a few seconds. The solubilized samples were centrifuged at 12000 × *g* for 10 min at 4°C to eliminate any insolubilized material and the supernatant was fractionated by ultracentrifugation at 41000 × *g* for 14 h at 4°C (Hitachi Preparative Ultracentrifuge CP90WX). The fractionation was done on a linear sucrose gradient containing 0.65 M sucrose, 0.008% (w/v) α-DM and 10 mM HEPES pH 7.5 formed directly in an ultracentrifuge tube by freezing at −80°C and thawing at 4°C. The band containing LHCII was carefully harvested using a syringe and then stored at −80°C. Before each experiment, PSII membranes or LHCII were exposed to continuous white light (1000 μmol photons m^-2^ s^-1^) at room temperature to induce high-light stress for the period mentioned in the respective figure legends. Light exposure was performed using a mercury-xenon lamp with a light guide (LIGHTNINGCURE Spot light source LC8, Hamamatsu, Japan). In some experiments, samples were illuminated in presence of either diphenylcarbazide (DPC) or desferal

### Electron paramagnetic resonance spectroscopy

Detection of superoxide anion radical (O_2_^•−^), hydroxyl radical (HO^•^) and ^1^O_2_ was performed using an electron paramagnetic resonance spectrometer (MiniScope MS400, Magnettech GmbH, Berlin, Germany). For O_2_^•−^ detection, a spin trap, EMPO, 5-(ethoxycarbonyl)-5-methyl-1-pyrroline N-oxide (Alexis Biochemicals, Lausen, Switzerland) was used. PSII membranes deprived of Mn_4_O_5_Ca complex (200 μg Chl) were exposed to high light (1000 μmol photons m^-2^ s^-1^) in presence of 50 mM EMPO, 50μM desferal and 40 mM MES buffer (pH 6.5). For HO^•^ detection, POBN/ethanol system was used. PSII membranes deprived of Mn_4_O_5_Ca complex (200 μg Chl) were exposed to high light (1000 μmol photons m^-2^ s^-1^) in presence of 50 mM POBN, 170 mM ethanol and 40 mM MES buffer (pH 6.5). After high light exposure, the sample was immediately transferred into a glass capillary tube (Blaubrand^®^ intraMARK, Brand, Germany) and EPR spectra were collected at room temperature. For ^1^O_2_ detection, a hydrophilic diamagnetic compound TMPD (2, 2, 6, 6-tetramethyl-4-piperidone, Sigma-Aldrich, USA) was used. TMPD was purified twice by vacuum distillation to reduce impurity from TMPD EPR signal. PSII deprived of Mn_4_O_5_Ca complex (200 μg Chl) were exposed to high light (1000 μmol photons m^-2^ s^-1^) in the presence of 50 mM TMPD and 40 mM MES buffer (pH 6.5). After high light exposure, the sample was centrifuged at 2000 x *g* for 1 min to separate TEMPONE from PSII membranes to prevent the oxidation of TEMPONE by non-specific oxidizing components. After centrifugation, the upper phase was immediately transferred into a glass capillary tube and EPR spectra were collected at room temperature. Signal intensity was calculated from the height of the central peak of EPR spectrum. EPR conditions were as follows: microwave power, 10 mW; modulation amplitude, 1 G; modulation frequency, 100 kHz; sweep width, 100 G; scan rate, 1.62 G s^-1^.

### SDS-PAGE and immunoblotting

To detect protein radical in PSII membranes deprived of Mn_4_O_5_Ca complex, immuno-spin trapping technique was used. PSII membranes deprived of Mn_4_O_5_Ca complex (10 μg Chl) were exposed to highlight for 30 min in the presence of 50 mM DMPO (5,5-dimethyl-1-pyrroline N-oxide). After high light exposure, proteins were extracted using DTT (dithiothreitol) protein extraction buffer, heating at 60°C dry bath for 30 min and centrifuged at 20000 x *g* for 5 min at 4°C. Supernatant was loaded into well and SDS-PAGE (sodium dodecyl sulfate- polyacrylamide gel electrophoresis) was run using a tris-tricine system at constant current (30 mA) according to the protocol described by Schagger[32]. Electrophoretic transfer to a nitrocellulose membrane (50 mA constant current for 1 h) was performed using a blotter (Trans-Blot SD, Semi-dry transfer cell, Bio-Rad, USA). The membrane was kept overnight at 4°C for blocking in 5% BSA prepared in phosphate buffered saline-tween 20 (PBST; pH 7.4). Next day, the membrane was transferred onto a shaker and left in the blocking solution for 30 min. All successive steps were performed on a shaker at room temperature. After blocking, the membrane was subjected to rabbit polyclonal anti-DMPO nitrone adduct antibody (1:5000, Abcam) for P^•^ detection. For identification of band near 32 kDa, anti-D1 and anti-D2 protein antibodies were used (Anti-D1, 1:15000, Anti-D2, 1:3000, Agrisera). For identification of bands near 43 and 47 kDa, anti-CP43 and anti-CP47 antibodies were used (Anti-CP43, 1:3000, Anti-CP47, 1:3000, Agrisera). The membranes were incubated with antibody for 1 h. After antibody treatment, the membrane was washed thrice with PBST for 10 min each and treated with horse radish peroxidase conjugated anti-rabbit IgG (1:10000) for 1 h. After this treatment, the membrane was again washed thrice with PBST for 10 min each and the bands were visualized using luminol as a chemiluminescent probe (Amersham Imager 600, GE Health Care Europe GmbH, Freiburg, Germany). The size of bands was determined using a protein ladder (PageRuler^™^ Prestained Protein Ladder, 10 to 180 kDa, Thermo Scientific, Lithuania).

### Fluorescence spectroscopy

Protein hydroperoxide was measured with a fluorescence spectrophotometer (F-4500, Hitachi, Tokyo, Japan) using a fluorescent probe Spy-LHP [33, 34]. PSII membranes deprived of Mn_4_O_5_Ca complex (50 μg Chl ml^-1^) were exposed to high light (1000 μmol photons m^-2^ s^-1^) for 30 min at 25°C. After high light exposure, PSII membranes deprived of Mn_4_O_5_Ca complex (5 μg Chl ml^-1^) were added to 2.5 μM Spy-LHP (Dojindo Laboratories, Japan) and incubated at 37°C for 30 min. After incubation, all the samples were centrifuged at 12000 x *g* for 2 min at 4°C and the supernatant containing SPY-LHPox was used for fluorescence measurement. Fluorescence emission spectrum wasmeasured at a spectral range between 530–620 nm (excitation wavelength, 524 nm). The spectral slit-width for excitation and emission monochromator was 5 nm. The fluorescence intensity at 538 nm was used for quantification of protein hydroperoxide formation.

### Two-dimensional ultra-weak photon emission

Two-dimensional imaging of ultra-weak photon emission was measured in PSII membranes deprived of Mn_4_O_5_Ca complex using highly sensitive charge coupled device (CCD) camera. All samples were dark-adapted for 30 min to eliminate any interference by delayed luminescence and measurements were performed in an experimental darkroom to restrict diffusion of light from the external light source. CCD camera VersArray 1300B (Princeton Instruments, Trenton, NJ, USA) with the spectral sensitivity of 350–1000 nm and almost 90% quantum efficiency in the visible range of the spectrum was used for two-dimensional photon emission imaging. A bandpass filter in the spectral range of 340–530 nm (Schott & Gen., Jena, Germany) was mounted on the objective lens of the CCD camera to eliminate the emission contributed by the chlorophylls. For reduction of dark current, CCD camera was cooled down to -104°C using a liquid-nitrogen cooling system. The data correction was made by subtracting the background noise before every measurement. The measurements were done under following parameters: scan rate, 100 kHz; gain, 2; accumulation time, 30 min [35].

## Results

### Detection of superoxide anion and hydroxyl radicals by EPR spintrapping

**Fig 1:**
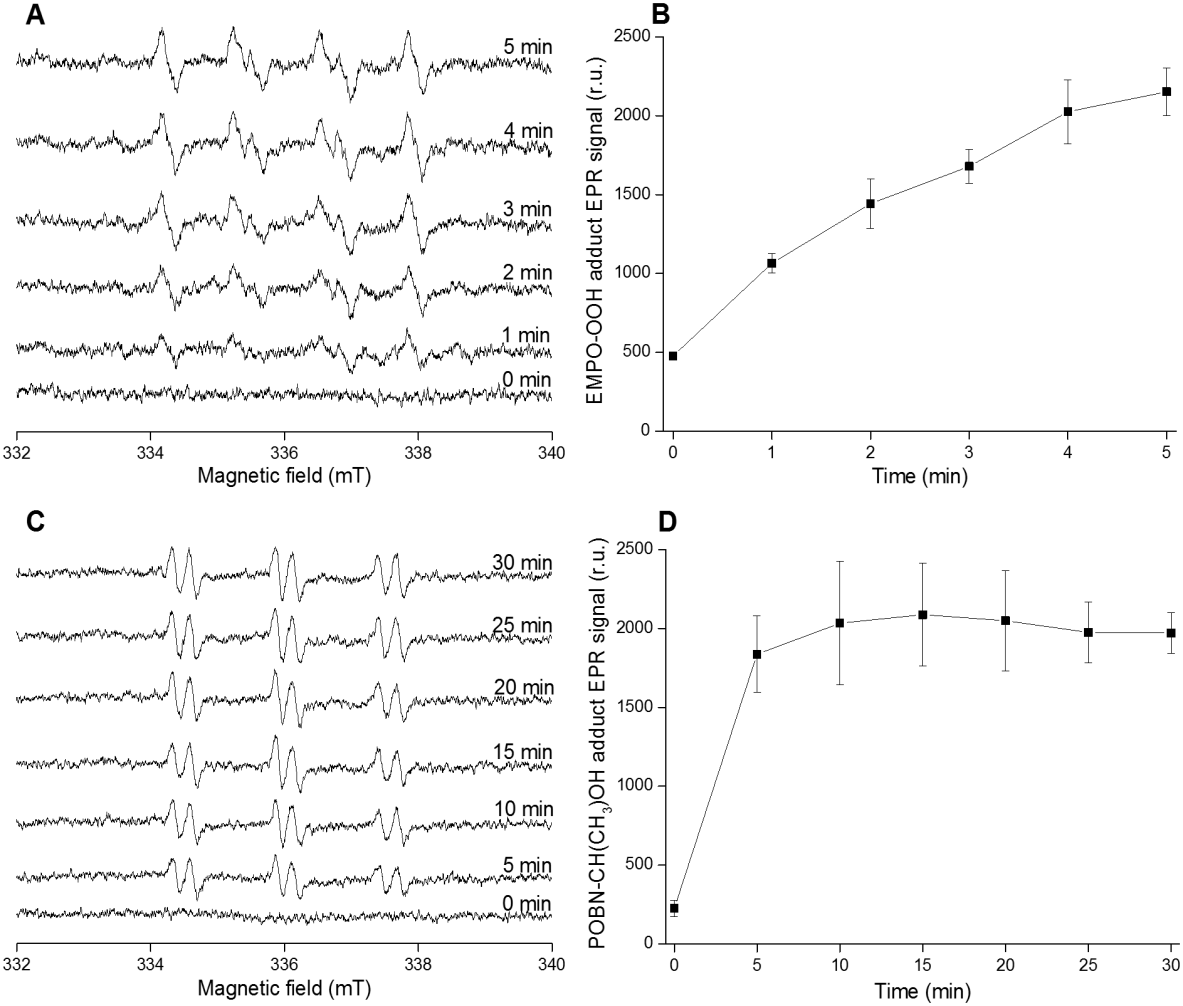
Superoxide anion and hydroxyl radical detection in PSII membranes deprived of Mn_4_O_5_Ca complex using EPR spintrapping spectroscopy. EMPO-OOH adduct EPR spectra [A], time dependence of EMPO-OOH adduct EPR signal [B], POBN-CH(CH_3_)OH adduct EPR spectra [C] and time dependence of POBN-CH(CH_3_)OH adduct EPR signal [D]. PSII membranes deprived of Mn_4_O_5_Ca complex (200 μg Chl) were exposed to high light (1000 μmol photons m^-2^ s^-1^) for the period mentioned in the figure. Superoxide anion radical spin trapping was accomplished in presence of 50 mM EMPO, 50 μM desferal and 40 mM MES buffer (pH 6.5). Hydroxyl radical spin trapping was performed in presence of 50 mM POBN, 170 mM ethanol and 40 mM MES buffer (pH 6.5). Data represent mean ± SD of three experiments.

To monitor O_2_^•−^ and HO^•^ formation in PSII membranes deprived of Mn_4_O_5_Ca complex EPR spintrapping spectroscopy was used. The spintrapping of O_2_^•−^ was accomplished in the presence of EMPO which is known to react with O_2_^•−^ forming the spin trap-superoxide (EMPO-OOH) adducts [36]. No EMPO-OOH adduct EPR signal was observed when EMPO spin trap was added to PSII membranes deprived of Mn_4_O_5_Ca complex in the dark (Fig 1A). Exposure of PSII membranes deprived of Mn_4_O_5_Ca complex to high light resulted in the appearance of EMPO-OOH adduct EPR spectra that exhibited fours peaks and hyperfine splitting characteristics of EMPO-OOH adduct. Due to the instability of EMPO-OOH adduct (half-life of 8 min), EMPO-OOH adduct EPR signal was measured upto 5 min. Time dependence of EMPO-OOH adduct EPR signal showed that EMPO-OOH adduct EPR signal increases with exposure time (Fig 1B). The spintrapping of HO^•^ was accomplished by POBN/ethanol system. In this system, oxidation of ethanol by HO^•^ forms 1-hydroxyethyl radical [CH(CH_3_)HO^•^] which reacts with POBN forming paramagnetic POBN-hydroxyethyl radical [POBN-CH(CH_3_)OH] adduct detectable by EPR [37]. In the dark, no POBN-CH(CH_3_)OH adduct EPR spectrum was observed, whereas, after exposure to light, POBN-CH(CH_3_)OH adduct EPR spectra were formed (Fig 1C). Time dependence of POBN-CH(CH_3_)OH adduct EPR signal showed that POBN-CH(CH_3_)OH adduct EPR signal increased significantly within 5 min illumination (Fig 1D). To explore, whether HO^•^ formation occurs in the PSII antenna complex, POBN-CH(CH_3_)OH adduct EPR spectra were measured in LHCII complex isolated from PSII membranes deprived of Mn_4_O_5_Ca complex. S1(A) and S1(B) Fig shows that POBN-CH(CH_3_)OH adduct EPR signal increased when the isolated LHCII complex was exposed to high light. These results indicate that exposure of PSII membranes deprived of Mn_4_O_5_Ca complex to high light resulted in O_2_^•−^ and HO^•^ formation.

### Detection of protein radical by immuno-spin trapping

**Fig 2:**
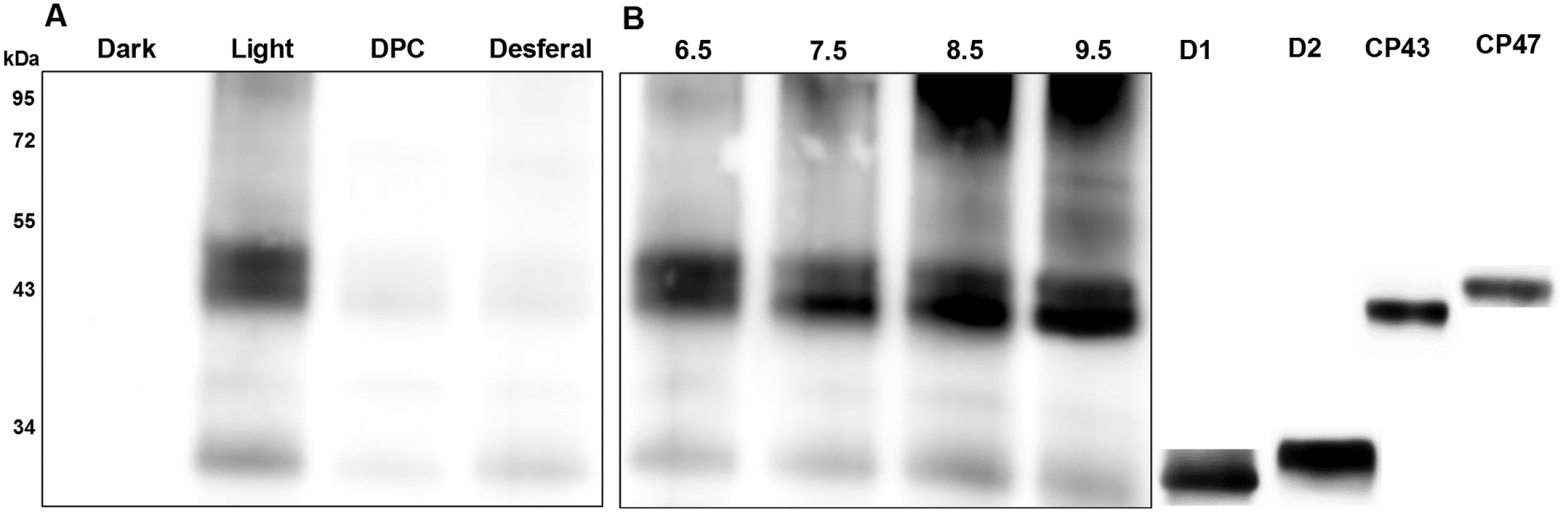
Protein radical detection in PSII membranes deprived of Mn_4_O_5_Ca complex using immuno-spin trapping technique. Blot images show effects of [A] DPC and desferal, and [B] high pH on P^•^ formation. Size of the marker is indicated on the left side and the identification of bands, which has been done on the same gel, is presented on the right side. For immuno-spin trapping detection of DMPO-protein nitrone adducts, anti DMPO-protein nitrone adduct antibody was used and for the identification of bands, respective antibodies were used. PSII membranes deprived of Mn_4_O_5_Ca complex (10 μg Chl) were exposed to high light (1000 μmol photons m^-2^ s^-1^) in presence of 50 mM DMPO and 40 mM MES buffer (pH 6.5) for 30 min. In some experiments, 500 μM DPC or 50 μM desferal were added to the sample before illumination. For high pH effect, PSII membranes deprived of Mn_4_O_5_Ca complex were exposed to high light in buffers of different pH (40 mM HEPES buffer pH 7.5, 40 mM HEPES buffer 8.5 and40 mM CAPSO buffer pH 9.5). These blots are the representative images of three experiments.

Detection of P^•^ was performed by immuno-spin trapping using DMPO as a spintrap. In this method, DMPO spin trap interacts with P^•^ forming DMPO-protein nitrone adduct which is detected using standard immunological techniques with antibodies raised against the nitrone of DMPO (anti-DMPO nitrone adduct antibody) [38]. Fig 2 shows anti-DMPO blots observed in PSII membranes deprived of Mn_4_O_5_Ca complex. In the dark, no band appeared, whereas after exposure to high light, anti-DMPO bands of 32 kDa and 43 kDa and 47 kDa appeared. The addition of DPC (an electron donor) or desferal (an iron chelator) suppressed the intensity of the anti-DMPO bands significantly (Fig 2A). When PSII membranes deprived of Mn_4_O_5_Ca complex were incubated at high pH, the intensity of 32 kDa, 43 kDa, and 47 kDa anti-DMPO bands was pronouncedly enhanced (Fig 2B). Identification of these anti-DMPO bands using respective antibodies confirmed that 32 kDa band represents D1 and D2 proteins, whereas 43 kDa and 47 kDa bands are assigned to CP43 and CP47 proteins. P^•^ formation in the CP43 protein was pronouncedly increased with the increase in pH while small changes were observed on other proteins (Fig 2B). These results indicate that exposure of PSII membranes deprived of Mn_4_O_5_Ca complex to high light results in P^•^ formation located mainly in the D1, D2, CP43 and CP47 proteins. Suppression of P^•^ formation by DPC and desferal indicates that P^•^ was formed by oxidation of proteins by P680^•+^ and HO^•^ while high pH promoted P^•^ formation.

### Detection of protein hydroperoxide by fluorescence spectroscopy

**Fig 3:**
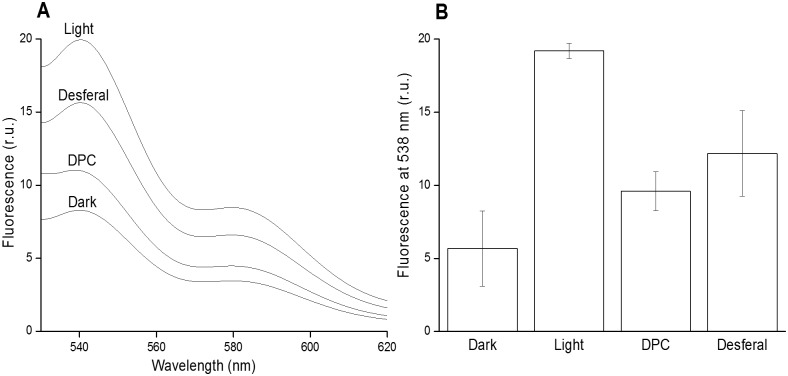
Protein hydroperoxide detection in PSII membranes deprived of Mn_4_O_5_Ca complex using fluorescence spectroscopy. Fluorescence emission spectra of Spy-LHPox after Spy-LHP being oxidized by hydroperoxide [A] and the fluorescence intensity at 538 nm [B]. PSII membranes deprived of Mn_4_O_5_Ca complex (50 μg Chl) were exposed to high light (1000 μmol photons m^-2^ s^-1^) in 40 mM MES buffer (pH 6.5) for 30 min. In some experiments, 500 μM DPC or 50 μM desferal were added to the sample before illumination. After light exposure, Spy-LHP (2.5 μM) was added to PSII membranes deprived of Mn_4_O_5_Ca complex and centrifuged after 30 min incubation at 37°C. Supernatant containing SPY-LHPox was used for fluorescence measurement. Data represent mean ± SD of three experiments.

Protein hydroperoxide (POOH) was detected by fluorescence spectroscopy using a fluorescent probe SPY-LHP. When a low fluorescence SPY-LHP is oxidized by POOH, a high fluorescence SPY-LHPox is formed [33, 34]. Fig 3 shows POOH formation in PSII membranes deprived of Mn_4_O_5_Ca complex. In the dark, a low SPY-LHP fluorescence was detected, whereas exposure of PSII membranes deprived of Mn_4_O_5_Ca complex to high light resulted in a high SPY-LHPox fluorescence. In presence of DPC or desferal, SPY-LHPox fluorescence was decreased (Fig 3A and 3B). These results indicate that POOH is formed after exposure of PSII membranes deprived of Mn_4_O_5_Ca complex to high light. Suppression of POOH formation by DPC and desferal reveals that POOH formation occurred from P^•^.

### Detection of protein triplet excited carbonyl by ultra-weak photon emission

**Fig 4:**
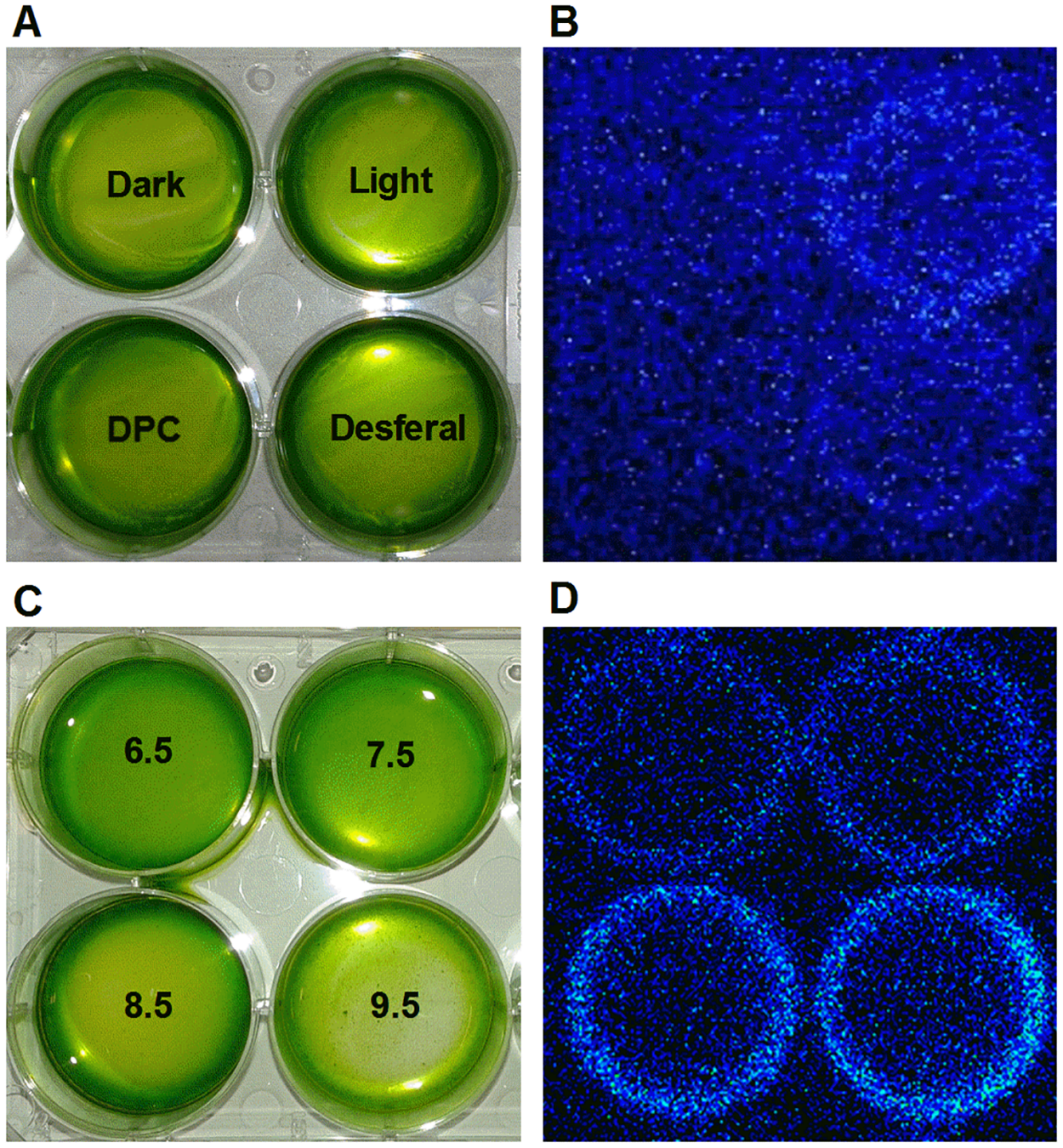
Detection of triplet carbonyl in PSII membranes deprived of Mn_4_O_5_Ca complex by ultra-weak photon emission using a CCD camera. The arrangement of samples in a petri-plate [A, C] and CCD images obtained after measurements [B, D]. PSII membranes deprived of Mn_4_O_5_Ca complex (300 μg Chl) were exposed to high light (1000 μmol photons m^-2^ s^-1^) in 40 mM MES buffer (pH 6.5) for 30 min. In some experiments, 500 μM DPC or 50 μM desferal were added to the sample before illumination. For high pH effect, PSII membranes deprived of Mn_4_O_5_Ca complex were exposed to high light in buffers of different pH (40 mM HEPES buffer pH 7.5, 40 mM HEPES buffer 8.5 and 40 mM CAPSO buffer pH 9.5). After high light exposure, all samples were dark-adapted for 30 min to eliminate any interference by delayed luminescence and the measurements were done in the spectral range of 340–530 nm to eliminate the emission contributed by chlorophylls. Data shown are the representative images of three experiments.

Protein triplet excited carbonyl (^3^P = O*) was monitored by the two-dimensional ultra-weak photon emission detected by CCD. ^3^P = O* is formed by decomposition of protein hydroperoxide under highly oxidizing conditions. Fig 4 shows ultra-weak photon emission measured in the blue-green region of the spectrum in PSII membranes deprived of Mn_4_O_5_Ca complex. In the dark, negligible ultra-weak photon emission was observed which increased many folds after exposure to high light (Fig 4A and 4B). When DPC was added to the sample, there was a strong decrease in the two-dimensional ultra-weak photon emission while the addition of desferal only decreased it slightly. The two-dimensional ultra-weak photon emission from ^3^P = O* was significantly increased when the samples were illuminated at high pH (Fig 4C and 4D). These results indicate that ^3^P = O* is formed in PSII membranes deprived of Mn_4_O_5_Ca complex after exposing it to high light. The decrease in ^3^P = O* formation by DPC or desferal indicates that ^3^P = O* was formed from POOH while ^3^P = O* formation was significantly enhanced at high pH.

### Detection of singlet oxygen by EPR spectroscopy

**Fig 5:**
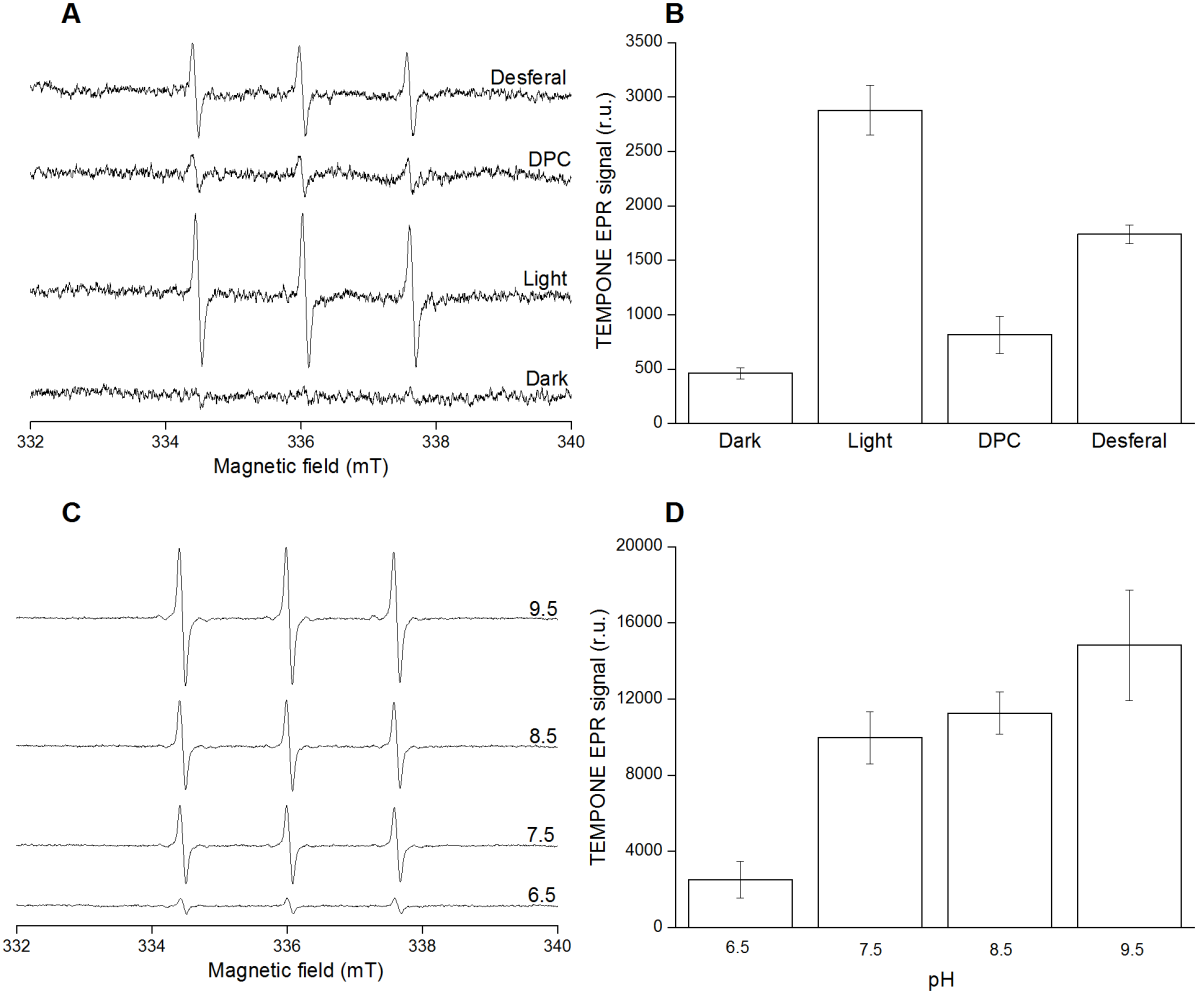
Singlet oxygen detection in PSII membranes deprived of Mn_4_O_5_Ca complex using EPR spectroscopy. TEMPONE EPR spectra [A, C] and a bar graph showing relative intensities of TEMPONE EPR signal [B, D]. PSII membranes deprived of Mn_4_O_5_Ca complex (200 μg Chl) were exposed to high light (1000 μmol photons m^-2^ s^-1^) in presence of 50 mM TMPD and 40 mM MES buffer (pH 6.5) for 30 min. In some experiments, 500 μM DPC or 50 μM desferal were added to the sample before illumination. For high pH effect, PSII membranes deprived of Mn_4_O_5_Ca complex were exposed to high light in buffers of different pH (40 mM HEPES buffer pH 7.5, 40 mM HEPES buffer 8.5 and40 mM CAPSO buffer pH 9.5). Data represent mean ± SD of three experiments.

Detection of ^1^O_2_ in PSII membranes deprived of Mn_4_O_5_Ca complex was accomplished by EPR spectroscopy. For this detection, a diamagnetic compound 2,2,6,6-tetramethyl-4-piperidone (TMPD) was used which after being oxidized by ^1^O_2_ forms a paramagnetic 2, 2, 6, 6-tetramethyl-4-piperidone-1-oxyl (TEMPONE) [39]. Fig 5 shows light-induced TEMPONE EPR spectra measured in PSII membranes deprived of Mn_4_O_5_Ca complex. The slight TEMPONE EPR signal observed in the dark was due to the residual impurity of TMPD, whereas TEMPONE EPR spectra were formed after exposure of the sample to light (Fig 5A and 5B). When DPC or desferal were added to the sample before light exposure, the TEMPONE EPR signal was suppressed (Fig 5A and 5B) while exposing samples to high light on high pH increased TEMPONE EPR signal significantly (Fig 5C and 5D). Similarly, when DPC or desferal were added to isolated LHCII complex before illumination the TEMPONE EPR signal was pronouncedly suppressed (S1 Fig). These results indicate that exposure of PSII membranes deprived of Mn_4_O_5_Ca complex to high light resulted in the formation of ^1^O_2_. Prevention of ^1^O_2_ formation by DPC or desferal indicates that ^1^O_2_ formation occurred from ^3^P = O* while ^1^O_2_ formation was enhanced at high pH.

## Discussion

Photosynthetic light reactions in PSII comprising of energy transfer and electron transport are associated with the ^1^O_2_ formed by triplet-triplet energy transfer from ^3^Chl* to O_2_ (Type II photosensitization reaction). Formation of ^3^Chl* is caused by the change in orientation of spin of an excited electron in the PSII antenna complex and the recombination of triplet radical pair ^3^[P680^•+^Pheo^•−^] in the PSII reaction center. Apart from the ^1^O_2_ formation by energy transfer from ^3^Chl* to O_2_, it was previously evidenced that ^1^O_2_ is formed by lipid peroxidation in PSII membranes deprived of Mn_4_O_5_Ca complex [15]. The authors demonstrated that lipid peroxidation initiated by highly oxidizing P680^•+^/TyrZ^•^ species is associated with L^•^ formation which interacts with O_2_ forming LOO^•^. A plausible mechanism for ^1^O_2_ formation was proposed to be recombination of LOO^•^ by Russell mechanism. It was proposed that POOH may also participate in reactions leading to the formation of ^1^O_2_; however, limited experimental evidence has been provided [40]. In this study, we have provided evidence on the ^1^O_2_ formation by the decomposition of POOH in PSII membranes deprived of Mn_4_O_5_Ca complex.

### Formation of highly oxidizing species

**Fig 6:**
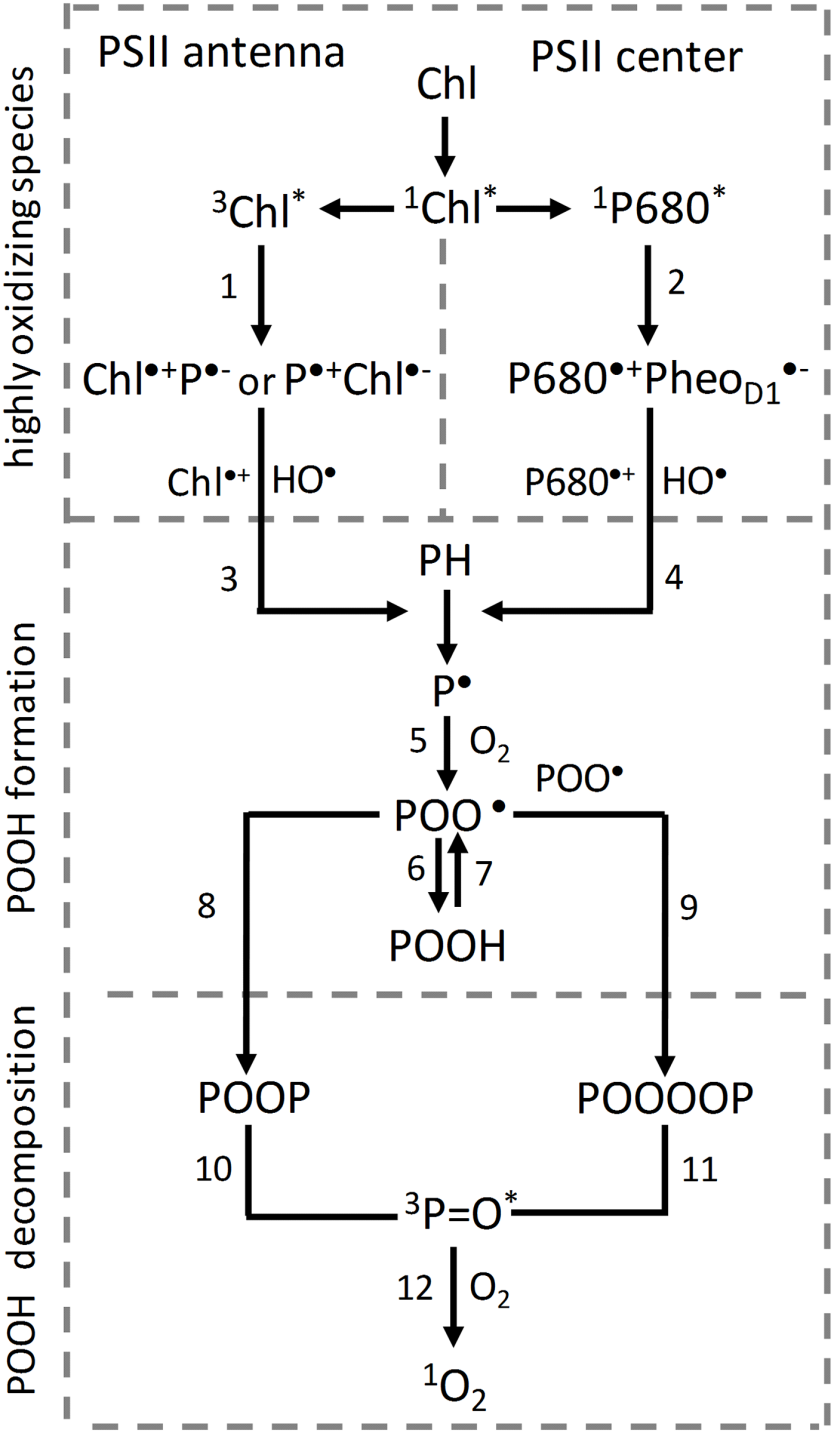
Decomposition of protein hydroperoxide to singlet oxygen. Formation of [Chl^•+^P^•−^] or [P^•+^Chl^•−^] radical pairs by photosensitization reaction Type I in the PSII antenna complex (reactions 1) and [P680^•+^PheoD_1_^•−^] radical pair by charge separation in the PSII reaction center (reactions 2). P^•^ formation by protein oxidation by highly oxidizing species (Chl^•+^ and HO^•^) in the PSII antenna complex (reaction 3) and (P680^•+^ and HO^•^) in the PSII reaction center (reaction 4). POO^•^ formation by reaction of O_2_ with P^•^ (reaction 5). POOH formed by hydrogen abstraction from proteins by POO^•^ (reaction 6). Oxidation of POOH forms POO^•^ (reaction 7). Formation of POOP and POOOOP from POO^•^ by cyclization and recombination, respectively (reactions 8 and 9). ^3^P = O* formation by POOH decomposition of POOP and POOOOP (reactions 10 and 11). ^1^O_2_ formation by triplet-triplet energy transfer from ^3^P = O* to O_2_ (reaction 12).

Based on the data presented in this study, it is proposed that protein oxidation is initiated by highly oxidizing species formed by charge separation of excited chlorophylls (Type I photosensitization reaction). In the PSII antenna complex, both singlet (^1^Chl*) and triplet (^3^Chl*) excited chlorophylls might undergo charge separation. However, due to the longer lifetime of ^3^Chl* (milliseconds) as compared to ^1^Chl* (nanoseconds) [41], it is proposed that the charge separation of ^3^Chl* is more likely. It is suggested that charge separation of ^3^Chl* forms either chlorophyll cation (Chl^•+^) and protein anion (P^•−^) radicals or chlorophyll anion (Chl^•−^) and protein cation (P^•+^) radicals (Fig 6, reaction 1). In agreement with this proposal, it was previously assumed that exposure of phycobilisomes from Synechocystis PCC 6803 to high light forms phycocyanin cation radical [42]. As the standard redox potential of Chl^•+^/Chl redox couple is highly positive (E_0_´ = 810 mV, chl *a* in a polar solvent) [43], Chl^•+^ has the capability to oxidize nearby proteins forming P^•^. Due to highly negative standard redox potential of Chl/Chl^•−^ redox couple (E_0_´ = − 1120 mV, chl *a* in polar solvent) [43], Chl^•−^ has the ability to reduce O_2_ forming O_2_^•−^. EPR spin trapping data using EMPO spin trap confirmed that exposure of PSII membranes deprived of Mn_4_O_5_Ca complex to high light resulted in O_2_^•−^ (Fig 1A and 1B). Superoxide anion radical dismutates spontaneously or enzymatically to hydrogen peroxide (H_2_O_2_) which is subsequently reduced to HO^•^ via Fenton reaction as confirmed by EPR spin trapping data using POBN/ethanol system (Fig 1C and 1D). The observation that exposure of isolated LHCII complex to high light results in formation of HO^•^ (S1 Fig) confirmed that HO^•^ is formed by photosensitization reaction Type I. Due to the highly positive standard redox potential of the HO^•^/H_2_O redox couple (E_0_´(HO^•^/H_2_O) = 2.3 V, pH 7) [44], HO^•^ has high capability of abstracting hydrogen from adjacent proteins. In the PSII reaction center, ^1^P680* undergoes charge separation forming P680^•+^ and Pheo^•−^ [8] (Fig 6, reaction 2). Due to highly positive midpoint redox potential of P680^•+^/P680 redox couple (*E*m = 1250 mV, pH 7) [6], P680^•+^ is able to abstract electron from nearby TyrZ forming TyrZ^•^. Formation of TyrZ^•^ by highly oxidizing P680^•+^ in PSII membranes deprived of Mn_4_O_5_Ca complex was reported in previous work [45]. As TyrZ^•^/TyrZ redox couple has highly positive midpoint redox potential (*E*m = 1100–1200 mV, pH 7), it has the capability to oxidize adjacent proteins. As Pheo/Pheo^•−^ redox couple has highly negative midpoint redox potential (*E*m = − 610 mV, pH 7), Pheo^•−^ has the ability to reduce O_2_ forming O_2_^•−^ which is subsequently reduced to H_2_O_2_ and HO^•^. In agreement with this proposal, it has been previously reported that reduction of O_2_ by Pheo^•−^ in D1/D2/cyt *b*_559_ complexes, which lack Q_A_ forms O_2_^•−^ [46]. The observation that HO^•^ formation in PSII membranes deprived of Mn_4_O_5_Ca complex is higher compared to LHCII complex may suggest that Pheo^•−^ contribute to O_2_^•−^ formation (Fig 1C and 1D and S1 Fig).

### Formation of protein hydroperoxide

Immuno-spin trapping detection of P^•^ confirmed that exposure of PSII membranes deprived of Mn_4_O_5_Ca complex to high light caused P^•^ formation in the PSII antenna complex and PSII reaction center (Fig 2). It is proposed that P^•^ formation is caused by hydrogen abstraction from adjacent proteins by highly oxidizing species in the PSII antenna complex (Chl^•+^ and HO^•^) (Fig 6, reaction 3) and the PSII reaction center (P680^•+^ and HO^•^) (Fig 6, reaction 4). In the PSII antenna complex, P^•^ is formed in the light-harvesting complex of PSII (LHCII) and the core antenna complex of PSII (CP43 and CP47) (Fig 2). The observation that reduction of highly oxidizing species by DPC prevented P^•^ formation completely in the LHCII, CP43 and CP47 proteins suggests that PSII antenna proteins are oxidized predominantly by Chl^•+^ (Fig 2A). When highly oxidizing HO^•^ was eliminated by desferal, P^•^ formation was partially prevented indicating that PSII antenna proteins are partially oxidized by HO^•^ (Fig 2A). It has also been reported that HO^•^ not only causes oxidation of PSII proteins but also interferes with the repair of PSII [47–49]. In the PSII reaction center, elimination of highly oxidizing P680^•+^ almost completely prevented P^•^ formation in the D1 and D2 proteins. Interestingly, P^•^ formation was pronouncedly enhanced in the CP43 proteins at high pH, whereas P^•^ formation in the D1 and D2 proteins was not affected. In agreement with this observation, it was shown that light-induced degradation of the CP43 protein in PSII membranes deprived of Mn_4_O_5_Ca complex is enhanced at high pH [50]. The authors showed that loss of CP43 protein occurs even in the absence of O_2_, although the rate of CP43 loss was lower than that under aerobic conditions.

The reaction of P^•^ with O_2_ is proposed to form POO^•^ (Fig 6, reaction 5). Due to the highly positive standard redox potential of the POO^•^/POOH redox couple (E_0_´ (POO^•^/POOH) = 1000 mV, pH 7), POO^•^ has the capability to abstract hydrogen from surrounding proteins to form another P^•^ and POOH [17] (Fig 6, reaction 6). Fluorescence spectroscopy using a fluorescent probe SPY-LHP showed that exposure of PSII membranes deprived of Mn_4_O_5_Ca complex to high light caused POOH formation (Fig 3). It was previously demonstrated that POOH and LOOH formed from bound lipid (associated with proteins) are produced in the initial phase of illumination, whereas LOOH formed from free-moving lipids (bulk lipids) are generated in the later phase of illumination [19]. The observation that removal of highly oxidizing species decreased POOH formation (Fig 3A and 3B) suggests that formation of POOH is initiated by Chl^•+^ and P680^•+^. It is generally accepted that POOH is stable; however, under highly oxidizing conditions, POOH is oxidized to POO^•^ (Fig 6, reaction 7).

### ^1^O_2_ formation by decomposition of protein hydroperoxide

It is proposed that POO^•^ either undergoes cyclization to form a cyclic intermediate dioxetane (POOP) (Fig 6, reaction 8) or recombines with another POO^•^ forming a linear intermediate tetroxide (POOOOP) (Fig 6, reaction 9). Decomposition of these high-energy intermediates (POOP and POOOOP) forms ^3^P = O* (Fig 6, reactions 10 and 11). Detection of ultra-weak photon emission in PSII membranes deprived of Mn_4_O_5_Ca complex suggests ^3^P = O* formation (Fig 4A and 4B). The observation that removal of highly oxidizing species decreased ^3^P = O* formation suggests that ^3^P = O* is initiated by Chl^•+^ and P680^•+^ (Fig 4A and 4B). In agreement with enhancement in P^•^ formation at high pH, ^3^P = O* formation was pronouncedly increased at high pH (Fig 4C and 4D). Triplet-triplet energy transfer from ^3^P = O* to O_2_ forms ^1^O_2_ (Fig 6, reactions 12) (Fig 5). EPR spectroscopy using TMPD confirmed that exposure of PSII membranes deprived of Mn_4_O_5_Ca complex to high light resulted in ^1^O_2_ (Fig 5A and 5B). In agreement with this proposal, it was demonstrated using ^18^O-labeled linoleic acid hydroperoxide and mass-spectrometry analysis that under oxidizing conditions with hypochlorous acid, oxygen atoms in LOOH serve as sources of ^1^O_2_ [51, 52]. More recently, it has been demonstrated that ^1^O_2_ is generated by triplet-triplet energy transfer from triplet excited acetone formed chemically and enzymatically [53]. The observation that removal of highly oxidizing species suppressed ^1^O_2_ formation pronouncedly (Fig 5A and 5B and S1C and S1D Fig) suggests that ^1^O_2_ formation is initiated by Chl^•+^ and P680^•+^. Based on the observation that DPC suppressed ^1^O_2_ formation compared to desferal, it is suggested that chlorophyll cation radicals (Chl^•+^ and P680^•+^) are major oxidant as compared to HO^•^. In agreement with this proposal, it was shown that the excited photosensitizer forms more commonly cation radical rather than anion radical [54]. Based on these considerations, it is proposed that ^1^O_2_ is formed by energy transfer from ^3^P = O* formed by Type I photosensitization reaction rather than from ^3^Chl* formed by Type II photosensitization reaction. This proposal is supported by the observation that ^1^O_2_ formation is enhanced at high pH (Fig 5C and 5D) in a similar manner as ^3^P = O* formation (Fig 4C and 4D). It has to be pointed out that the residual ^1^O_2_ formation observed in the presence of DPC and desferal corresponds to ^1^O_2_ formation formed by Type II photosensitization reaction.

## Conclusion

Data presented in this study provide evidence on the ^1^O_2_ formation by excitation energy transfer from ^3^P = O* to O_2_ which represents an alternative way to the well-known ^1^O_2_ formation by energy transfer from ^3^Chl* to O_2_ by Type II photosensitization reaction. It is demonstrated that ^3^P = O* is formed by protein oxidation initiated by Type I photosensitization reaction. It is assumed that Type II photosensitization reaction is a major source of ^1^O_2_ formation in PSII with Mn_4_O_5_Ca complex, whereas Type I photosensitization reaction more pronouncedly contributes to the overall ^1^O_2_ formation in PSII membranes deprived of Mn_4_O_5_Ca complex.

## Supporting information

S1 Fig Hydroxyl radical and singlet oxygen detection in isolated LHCII using EPR spectroscopy. POBN-CH(CH_3_)OH adduct EPR spectra [A], time dependence of POBN-CH(CH_3_)OH adduct EPR signal [B], TEMPONE EPR spectra [C], and bar graph showing relative intensity of TEMPONE EPR signal [D]. LHCII (20 μg Chl) were exposed to high light (1000 μmol photons m^-2^ s^-1^) for period mentioned in the figure. Hydroxyl radical measurement was accomplished using 50 mM POBN, 170 mM ethanol and 40 mM MES buffer (pH 6.5) while 50 mM TMPD and 40 mM MES buffer (pH 6.5) were used for singlet oxygen measurement. In some of the singlet oxygen experiments, 500 μM DPC or 50 μM desferal were added to the sample before illumination. Data represent mean ± SD of three experiments.
(PDF)
