# Supplementary figures and images for: Formation of singlet oxygen by decomposition of protein hydroperoxide in photosystem II
Source: PLoS One. 2017 Jul 21;12(7):e0181732. doi: 10.1371/journal.pone.0181732 (PMC5521840; doi:10.1371/journal.pone.0181732)

S1 Fig

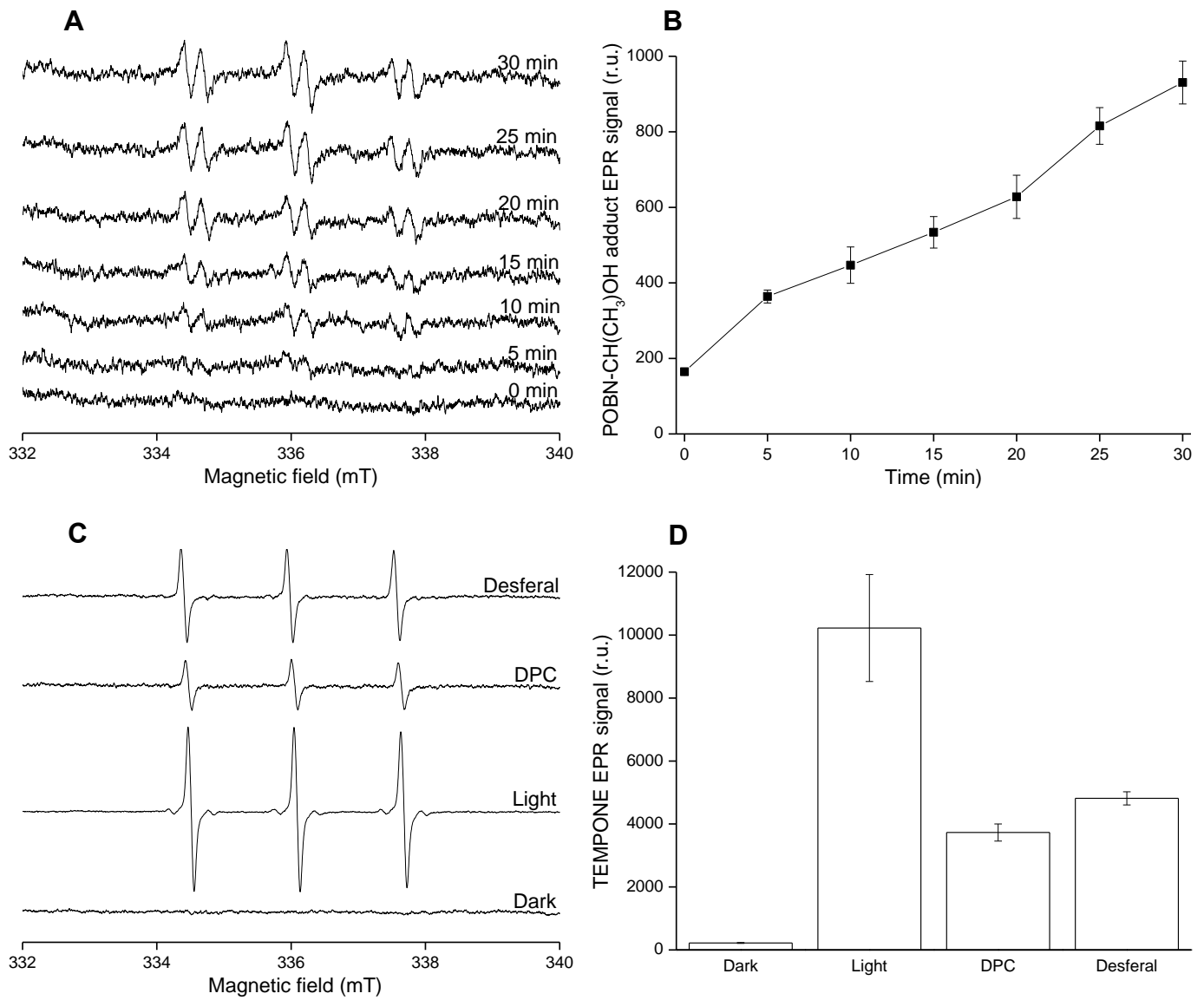

Supplement: S1 Fig — POBN-CH(CH3)OH adduct EPR spectra [A], time dependence of POBN-CH(CH3)OH adduct EPR signal [B], TEMPONE EPR spectra [C], and bar graph showing relative intensity of TEMPONE EPR signal [D]. LHCII (20 μg Chl) were exposed to high light (1000 μmol photons m-2 s-1) for period mentioned in the figure. Hydroxyl radical measurement was accomplished using 50 mM POBN, 170 mM ethanol and 40 mM MES buffer (pH 6.5) while 50 mM TMPD and 40 mM MES buffer (pH 6.5) were used for singlet oxygen measurement. In some of the singlet oxygen experiments, 500 μM DPC or 50 μM desferal were added to the sample before illumination. Data represent mean ± SD of three experiments. (PDF) [file pone.0181732.s001.pdf]
